# Preoperative low skeletal muscle mass index assessed using L3-CT as a prognostic marker of clinical outcomes in pancreatic cancer patients undergoing surgery: a systematic review and meta-analysis

**DOI:** 10.1097/JS9.0000000000000989

**Published:** 2023-12-11

**Authors:** Pauline Raoul, Marco Cintoni, Alessandro Coppola, Sergio Alfieri, Giampaolo Tortora, Antonio Gasbarrini, Maria Cristina Mele, Emanuele Rinninella

**Affiliations:** a Clinical Nutrition Unit; b Digestive Surgery Unit; c Medical Oncology Unit; d Digestive Disease Center (CEMAD), Department of Medical and Abdominal Surgery and Endocrine-Metabolic Sciences; Fondazione Policlinico Universitario A. Gemelli IRCCS; e Research Center in Human Nutrition; f Department of Translational Medicine and Surgery Catholic University of the Sacred Heart; g Department of Surgical Sciences, University of Rome La Sapienza, Rome, Italy

**Keywords:** mortality, nutrition, overall survival, pancreatic cancer, sarcopenia, skeletal muscle index (SMI)

## Abstract

**Background:**

Reduction in muscle mass can be routinely quantified using computed tomography (CT) of the third lumbar vertebra (L3) during a curative pancreatic cancer (PC) course. This systematic review and meta-analysis aimed to assess the association between preoperative low skeletal muscle index (SMI) measured by L3-CT and postoperative clinical outcomes in PC resectable patients.

**Methods:**

Three electronic databases (PubMed, Web of Science, and Scopus) were searched for articles published through May 2023. Duplicate titles and abstracts, full-text screening, and data extraction were performed. A meta-analysis was performed for overall survival (OS), recurrence-free survival (RFS), postoperative pancreatic fistula (POPF), morbidity, and postoperative length of stay (P-LOS). The risk of bias was assessed.

**Results:**

A total of 2942 patients with PC from 11 studies were identified. Preoperative low SMI was found in 50.9% of PC resectable patients. Preoperative low SMI was significantly associated with adjusted OS (adjusted hazard ratio, 1.52; 95% CI 1.25–1.86, *P*< 0.0001). No significant associations were found between preoperative low SMI and RFS, number of POPF, significant morbidity, and P-LOS (*P*>0.05).

**Conclusions:**

SMI should be evaluated in a timely manner as a predictor of OS in PC resectable patients. Studies assessing nutritional protocols for maintaining/increasing skeletal muscle mass are required to develop a personalized nutritional approach to improve clinical outcomes.

## Introduction

Highlights

- Pancreatic cancer (PC) is one of the most frequent and deadly cancers worldwide.
- In pancreatic surgery, the scope for improvement includes high postoperative morbidity and the translation of surgical success into long-term survival.
- Sarcopenia has been identified as a negative prognostic factor influencing morbidity and mortality in patients with resectable PC.
- Computed tomography performed by patients with PC for the pancreatic disease stage can be used for the analysis of body composition, particularly for skeletal muscle mass quantity assessment.
- Skeletal muscle mass index derived from computed tomography scan at the third lumbar vertebra significantly correlates with worse overall survival.
- Skeletal muscle mass index should be evaluated in a timely manner as a predictor of overall survival in PC resectable patients to prompt individualized nutritional support since from a pre-intervention phase.

Pancreatic cancer (PC) is one of the most frequent and deadly cancers worldwide with a 5-year survival rate of 6–10%^1^. Patients with PC are typically diagnosed with advanced disease owing to a lack of specific and recognized symptoms when the cancer is still localized. Screening for PC in asymptomatic adult populations is not effective at the present time^2^. Even though the efficacy of therapeutic approaches over the past decade has improved, the challenge remains to optimize results by personalizing therapy since diagnosis^3^. Less than 20% will are eligible for resectable surgery^4^. In pancreatic surgery, the scope for improvement includes high postoperative morbidity and the inability to uniformly translate surgical success into long-term survival^4^. Recently, sarcopenia has been identified as a negative prognostic factor influencing morbidity and mortality in patients with resectable PC. Sarcopenia is characterized by low muscle strength, quantity or quality^5^. Several methods and instruments, such as bioelectrical impedance analysis (BIA), anthropometry, and dual-energy X-ray absorptiometry (DXA), have been used to determine the quantity and quality of muscle mass and its impact on a patient’s disease^6^. Computed tomography (CT) performed by patients with PC for the pancreatic disease stage is also used for the analysis of body composition, particularly for skeletal muscle mass quantity assessment^7^. The skeletal muscle index (SMI), which is calculated using the skeletal muscle area (SMA)/height^2^ obtained from a CT scan image of muscle mass at the third lumbar vertebra (L3), is a significant predictor of mortality in cancer populations^7–9^. Given the high number of methods used to assess sarcopenia, a systematic, large, and homogeneous synthesis of the scientific literature to identify a reliable tool in the preoperative phase is warranted. This systematic review and meta-analysis aimed to assess the reliability of assessing the low skeletal muscle index measured by L3-CT scan as a prognostic preoperative marker of clinical outcomes such as overall survival (OS), recurrence-free survival (RFS), postoperative pancreatic fistula (POPF), morbidity, postoperative length of stay (P-LOS), and 30-day mortality in patients with resectable PC.

## Methods

This paper has been reported in line with PRISMA (Preferred Reporting Items for Systematic Reviews and Meta-Analyses)^10^ and AMSTAR (Assessing the methodological quality of systematic reviews) Guidelines^11^, Supplemental Digital Content 1, http://links.lww.com/JS9/B524. The PRISMA statement checklist for reporting systematic reviews is presented in Table S1, Supplemental Digital Content 2, http://links.lww.com/JS9/B525.

### Eligibility criteria

Studies that met the following criteria were included: (i) cohort prospective or retrospective studies, nested case-control studies, or case-control studies; (ii) studies including patients with PC undergoing pancreatic surgery, such as pancreaticoduodenectomy (PD), total pancreatectomy (TP), or distal pancreatectomy (DP); (iii) studies reporting a hazard ratio (HR) and 95% CI or Kaplan-Meier survival curves from which an HR could be calculated; (iv) studies using CT images to measure preoperative SMI at the L3 level; and (v) studies reporting preoperative low SMI as a dichotomous variable. SMI could be categorized with different sex-specific cut-off points such as those of 52.4 cm^2^/m^2^ for men and less than 38.5 cm^2^/m^2^ for women^12^.

The exclusion criteria were as follows: (1) studies including patients with other types of cancer, (2) reporting insufficient data for estimating the HR and 95% CI, (3) reporting low muscle mass measured by methods other than CT-SMI measurements, (4) reporting SMI only as a continuous variable, (5) studies not written in English, and (6) animal studies and case reports. There were no restrictions on publication date.

### Literature search strategy

Three electronic databases, MEDLINE (via PubMed), ISI Web of Science, and Scopus, were searched on 19 May, 2023. Databases were screened for search terms in the titles and abstracts. The search string for each database is presented in Suppl. data Table S2, Supplemental Digital Content 3, http://links.lww.com/JS9/B526. Reference lists were used to supplement the electronic search. Manual searches of the eligible articles were performed to identify additional relevant studies.

### Study selection

All articles generated from the electronic search were imported into Mendeley, a reference management software, and duplicates were removed. Three investigators (E.R., M.C., and P.R.) reviewed the titles and abstracts of all the identified studies to ascertain whether they were eligible for this systematic review based on our inclusion criteria. Discrepancies were resolved through discussions.

### Data extraction

Information was collected using an Excel template specifically developed for this study. Each full-text article was retrieved; articles deemed ineligible were excluded, and their reasoning was reported. Data extraction included study and sample patient details, cut-off defining low SMI for men and women, percentage of patients with preoperative low SMI, and HRs with associated 95% CIs or *P* values for each clinical outcome. HRs were extracted from multivariable and/or univariate analyses or estimated using Kaplan-Meier survival curves. Baseline clinical data of the patients (age, BMI, type of resection, operation time, and use of neoadjuvant or adjuvant chemotherapy) were collected. Survival outcomes, such as OS and RFS, as well as clinically relevant postoperative outcomes, such as significant morbidity, P-LOS, and 30-day mortality, have been reported.

### Exposure variables and outcomes

Muscle mass was measured using SMI and diagnosed by examination of the cross-sectional CT image at the L3 level. SMI was calculated as follows: [cross-sectional area of skeletal muscle] (cm^2^)/ height^2^ (m^2^). OS was defined as the time from the day of surgery to death from any cause. RFS was defined as the time from the date of surgery to the date of relapse or death from any cause. Clinical POPF was defined by the International Study Group on Pancreatic Fistula^13^. We focused on grades B or C because grade A was no longer considered a true pancreatic fistula. In particular, grade B requires a change in postoperative management; drains are either left in place for >3 weeks or repositioned through endoscopic or percutaneous procedures. Grade C refers to POPFs requiring reoperation or leading to single or multiple organ failure and/or mortality attributable to pancreatic fistulas. Significant morbidity was defined as a Clavien–Dindo score of ≥ 3^14^.

### Quality assessment

The quality of the studies was reviewed using the Newcastle–Ottawa quality assessment scale for case-control and cohort studies^15^. The quality score was calculated based on the following three categories: group selection, comparability between groups, and outcomes. A maximum of one star was awarded for each item in the group selection, outcome, and exposure assessment categories. A maximum of two stars can be awarded for comparability. Thus, nine points represented the highest methodological quality scores.

### Statistical analysis

The outcomes of this study were to estimate the pooled HR for the association between preoperative low SMI (assessed by CT scan at L3) and survival and surgical outcomes in patients with PC undergoing pancreatic surgery. When these data were not reported in the publications, we calculated the HR estimates and their 95% CIs using the survival probabilities in the Kaplan–Meier curve at specific time points according to the methods proposed by Parmar *et al*.^16,17^. For the continuous outcome P-LOS, the mean differences (MD) with 95% CIs were used to express the effect sizes. Odds ratios (ORs) and risk ratios were used for dichotomous variables, such as POPF and postoperative complications (morbidity). Statistical significance was set at *P* less than 0.05. The overall heterogeneity was assessed by measuring the inconsistency (I^2^) using the χ^2^ test. I^2^ values of 25%, 50%, and 75% represented cut-off points of low, moderate, and high degrees of heterogeneity, respectively, based on Cochrane’s rough guide for the interpretation of I^2^ ^18^. A random-effects model was used if heterogeneity between studies was high; otherwise, a fixed-effects model was used. Multivariate analyses were performed to evaluate the effects of various factors such as age, sex, BMI, Charlson comorbidities, and type of resection. The publication bias was evaluated through an asymmetry test as estimated from a funnel plot. The software RevMan v5.3; Nordic Cochrane Centre) was used for all statistical analyses.

## Results

### Study selection

**Figure 1:**
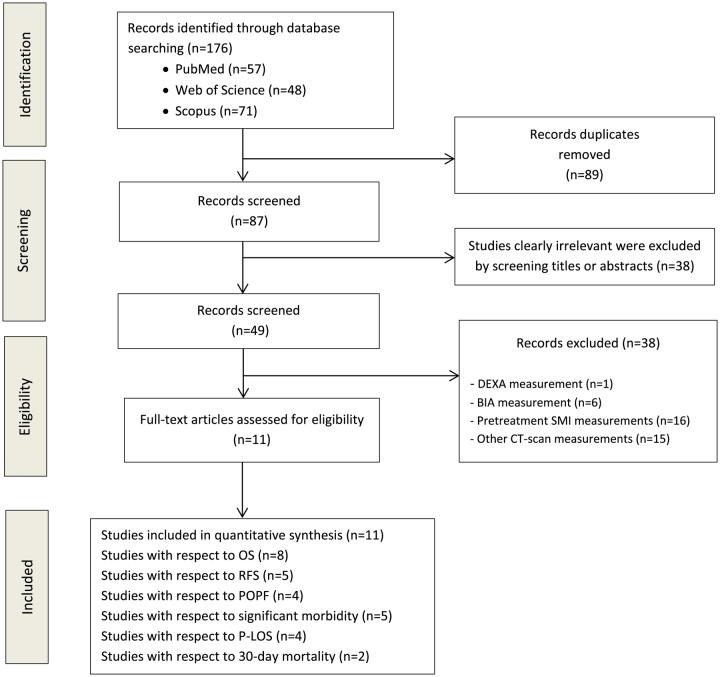
Preferred Reporting Items for Systematic Reviews and Meta-Analyses (PRISMA) flow diagram. BIA, body impedance analysis; CT, computed tomography; DEXA, dual-energy X-ray absorptiometry; OS, overall survival; P-LOS, postoperative length of hospital stay; POPF, postoperative pancreatic fistula; RFS, recurrence-free survival; SMI, skeletal muscle index.

One hundred seventy-six publications—of which 89 were duplicates, were initially identified, and 38 irrelevant studies were discarded. We excluded 38 studies that used criteria other than SMI from CT imaging to evaluate low muscle mass (Supplementary data Table S3, Supplemental Digital Content 4, http://links.lww.com/JS9/B527). Eleven studies met the inclusion criteria and were included in the systematic review. The flow diagram in (Fig. 1) displays the results of the literature search and study selection process.

### Study characteristics

Eleven studies^19–29^ included 2942 PC patients. Table 1 presents the main characteristics of the included studies. Patients were identified in retrospective studies in Japan (*n*=3)^20,21,29^, Italy (*n*=2)^19,27^, South Korea (*n*=2)^22,25^, China (*n*=1)^28^, the United States (*n*=1)^23^, Israel (*n*=1)^26^, and Austria (*n*=1)^24^. All the included studies were published in English between 2016^19^ and 2023^27–29^. All studies have a preoperative assessment. A total of 1498 patients were diagnosed with low preoperative SMI and 1444 patients with high preoperative SMI. Regarding the cut-off of SMI, out of 5 studies^19,20,24,27,28^ out of 11 used the cut-offs of Prado *et al*.^12^. In one study^21^, sex-specific cut-off values for SMI were established using receiver operating characteristic (ROC) curves. For the three studies, cut-off values were set at the lowest tertile^22,23^ or lowest quartile^26^ for SMI. For one study^25^, the cut-off of Moon *et al*.^30^. was applied, representing the standard values determined in a large cohort study conducted recently by a Korean national institution. Finally, one study used the sex-specific median SMI value as the cut-off^29^.

**Table 1:** Main characteristics of included studies.

Author, year	Country	No. patients	Cut-off value of low SMI for men (cm^2^/m^2^)	Cut-off value of low SMI for women (cm^2^/m^2^)	Preoperative low muscle mass rates (%)	Type of intervention	Postoperative outcomes	NOS score
Pecorelli *et al*.^19^	Italy	202	52.4	38.5	65	PD	60-day postoperative mortality Clinically relevant POPF Major complication (CDC grade _ III)	8
Ninomiya *et al*.^20^	Japan	265	52.4	38.5	64	PD, TP, DP	OS Major complication (CDC grade _ III)	8
Okumura *et al*.^21^	Japan	301	47.1	36.6	40	PD, DP, TP	OS RFS	8
Choi *et al*.^22^	South Korea	180	Lowest tertile 45.3	Lowest tertile 39.3	33		OS P-LOS	8
Sugimoto *et al*.^23^	United States	323	Lowest tertile 55.4	Lowest tertile 38.9	62	PD, DP, TP	OS	8
Gruber *et al*.^24^	Austria	133	52.4	38.5	59	PD, DP	OS RFS Clinically relevant POPF Major complication (CDC grade >III)	8
Ryu *et al*.^25^	South Korea	548	50.18	38.63	46	PD	Major complication (CDC grade > III) Clinically relevant POPF P-LOS	7
Rom *et al*.^26^	Israel	111	the lowest quartile	the lowest quartile	25	PD, DP	OS RFS	8
Menozzi *et al*.^27^	Italy	103	52.4	38.5	56.3	PD, DP, TP	Pancreatic fistula and biliary fistula Delayed Gastric Emptying Digestive Haemorrhage Clavien Score III IV Clavien Score V 30 days reintervention 30 days readmission	8
Shen *et al*.^28^	China	614	52.4	38.5	62	TP	OS RFS P-LOS	8
Masuda *et al*.^29^	Japan	162	41.9	36.6	50	ND	OS RFS Clinically relevant POPF Major complication (CDC grade >III) P-LOS	8

CDC; Clavien–Dindo classification; CT, computer tomography; DP, distal pancreatectomy; ND, not defined; NOS, Newcastle–Ottawa Scale; OS, overall survival; PD, pancreaticoduodenectomy; POPF, postoperative pancreatic fistula; P-LOS, postoperative length of hospital stay; RFS, recurrence-free survival; SMI, skeletal muscle index; TP, total pancreatectomy.

### Publication bias and study quality

The funnel plots (Suppl. data Figure S1 a-e, Supplemental Digital Content 5, http://links.lww.com/JS9/B528) are symmetrical around the summary estimate. Consequently, they did not report any publication bias. NOS scores for each study are presented in Table 1. The scores of each NOS item for each study are detailed in Table S4 (Suppl. Data, Supplemental Digital Content 6, http://links.lww.com/JS9/B529). All studies reached no less than seven stars, and the NOS score of eight stars was assigned to nine out of 11 studies concerning the representativeness and comparability of cohorts, assessment of outcome, and adequate follow-up of cohorts.

### Survival outcomes

#### Overall survival

**Figure 2:**
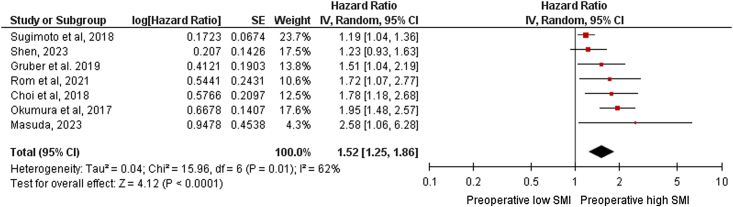
Forest plots of hazard ratios for adjusted overall survival (OS). IV, inverse variance; SE standard error.

A total of 2324 from seven studies^20,21,23,24,26,28,29^ were included in the univariate analysis of unadjusted HRs for OS. In comparison with high SMI patients, with low SMI patients reported a significantly worse OS (HR 1.49, 95% CI 1.08–2.04, *P*=0.01). The test for heterogeneity was significant (*P*=0.01; I^2^=87%). A total of 1824 patients from seven studies^21–24,26,28,29^ were included in the multivariate analysis of the adjusted HRs for OS. In comparison with high SMI patients, low SMI patients reported a significantly worse OS (HR, 1.52; 95% CI 1.25–1.86, *P*< 0.0001; Fig. 2). The test for heterogeneity was significant (*P*=0.01; I^2^=62%).

#### Recurrence-free survival (RFS)

RFS was reported in 5 studies^21,24,26,28,29^. Three studies^24,28,29^ did not show a significant association between low SMI and high RFS. Gruber and colleagues showed that low muscle mass did not impair RFS (15 months vs. 25 months, *P*=0.275). Masuda and colleagues demonstrated slightly higher RFS in low SMI PC patients, although the trends were not statistically significant (2-year RFS: 37.7% vs. 30.9%, *P* = 0.42). Another study^28^ showed that neither sarcopenia nor cachexia was associated with RFS in PC patients following radical excision (log-rank *P* > 0.05). However, two studies^21^ demonstrated a significant HR for RFS in multivariate analysis, with HR 1.60, 95% CI (1.18–2.16), *P*=0.002, and HR 1.8, 95% CI (1.1–2.8), *P*=0.01.

### Surgery outcomes

#### Postoperative pancreatic fistula

**Figure 3:**
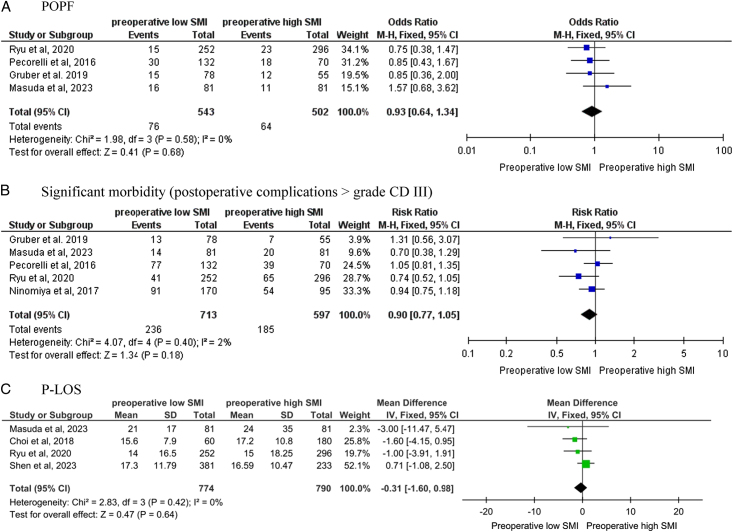
Forest plots assessing the association of skeletal muscle index (SMI) with postoperative pancreatic fistula (A), significant morbidity (B), and postoperative length of stay (C). CD, Clavien–Dindo; P-LOS, postoperative length of stay; POPF, postoperative pancreatic fistula; SD, standard deviation; SMI, skeletal muscle index.

Four studies^19,24,25,29^ including 1045 PC patients undergoing pancreatic surgery, reported the number of POPF in the low SMI vs. high SMI groups. A meta-analysis of these studies showed non-significant results between the two groups (OR, 0.93; 95% CI 0.64–1.34, *P*=0.68; Fig. 3A). A significant heterogeneity between studies was found (*P*=0.58, I2=0%).

#### Significant morbidity

Four studies^19,24,25,29^, including patients with PC undergoing pancreatic surgery, reported the number of significant morbidities (CDC grade ≥ 3) in the low SMI vs. high SMI groups. A meta-analysis of these studies showed non-significant results between the two groups (RR, 0.90; 95% CI 0.77–1.05, *P*=0.18; Fig. 3B). A significant heterogeneity between studies was found (*P*=0.40, I2=2%).

#### Postoperative length of stay

Four studies^22,25,28,29^ including 1464 patients undergoing pancreatectomy, reported the duration of P-LOS in days. A meta-analysis of these studies showed a shorter P-LOS in the higher muscle mass group (MD -0.31, 95% CI -1.60 to 0.98, Fig. 3C), although the results were not significant (*P*=0.64). A significant heterogeneity between studies was found (*P*=0.42, I2=0%).

#### Thirty-day mortality

Only two studies^25,29^ reported 30-day mortality as a surgical outcome. Both studies demonstrated non-significant differences between the low and high SMI groups (*P*>0.05).

## Discussion

This meta-analysis of more than 2900 patients evaluated preoperative muscle mass, assessed by L3-CT scan, as a prognostic marker in PC resectable patients undergoing surgery, showing that more than half of the patients (50.9%) had low muscle mass in the preoperative phase. Pooled adjusted analyses of data from more than 2000 PC patients found a significant association between low SMI and worse OS, a trend of increased RFS, P-LOS, and increased risk of significant postoperative morbidity and POPF, although non-significant.

Our results highlight the effect of reduced muscle mass on OS before pancreatic surgery, updating and confirming the results of another meta-analysis^31^. Thus, in the preoperative phase and as early as possible, nutritional interventions and physical activity are two crucial nonpharmacological approaches to contrast the reduction in muscle mass. Indeed, exercise, especially resistance exercise, has the benefit of improving muscle strength and mass, which helps improve the quality of life of patients with low SMI^32,33^. An adequate preoperative personalized nutrition program is the cornerstone of PC for sarcopenia treatment. However, the effect of nutritional interventions on muscle mass remains unclear. Currently, cancer consensus recommendations suggest a protein intake above 1.0 g/kg per day up to 2.0 g/kg per day for cancer patients undergoing surgery, although the ideal amount for some amino acids in isolation is yet to be determined^34^.

This study also demonstrated no significant association between low SMI before pancreatic surgery and postoperative clinical outcomes such as morbidity, risk of POPF, and LOS. Several previous studies and meta-analyses^31,35–37^ have demonstrated no significant difference in morbidity, which is consistent with our results. The significant impact of low muscle mass on postoperative short-term surgical outcomes varies across gastrointestinal cancer types. Indeed, in gastric and colorectal cancers, a significant association between preoperative sarcopenia and postoperative complications is demonstrated^38,39^. Regarding pancreatic surgery, it has been recently hypothesized that visceral fat could most likely influence patients’ complications, especially POPFs^40,41^, becoming clearly an important question for future research.

All included studies assessing the effect of disease-related sarcopenia on the surgical outcomes of patients with PC have only focused on the loss of muscle mass quantity, without measuring muscle strength or performance representing the main criteria of sarcopenia^5^. Consequently, further studies are needed to assess whether muscle function/quality, has a significant negative effect on surgical outcomes. The hand grip strength (HGS) test, widely used in community-dwelling people and outpatients, is quite useful and convenient for sarcopenia screening. However, the reliability of measuring HGS includes some technical issues, such as differences between measuring instruments^42^.

In this meta-analysis, only retrospective studies were included. Retrospective cohort studies evaluated cause-and-effect relationships between sarcopenia and outcomes, but due to their retrospective nature, these studies do not explain the underlying effects explaining the significance of these relationships. Malignancy-derived inflammation contributes to the genesis of sarcopenia in cancer patients^43^. Indeed, pro-inflammatory cytokines such as tumour necrosis factor-alpha, interleukin 1, and interleukin 6 lead to alterations in metabolic and endocrine pathways and have catabolic effects, leading to muscle loss^44^. Catabolic effects increase the need for certain amino acids which may be provided by the breakdown of skeletal muscle^45^. These mechanisms may increase tumour aggressiveness and lead to reduced survival^45,46^. In this context, various systematic reviews focusing on enhanced recovery after surgery (ERAS) programs or immunonutrition^47–49^ demonstrated that ERAS protocols, including the reduction of the preoperative fasting period (6 h for solids and 2 h for liquids) and oral carbohydrate loading 2 h before the intervention in non-diabetic patients, could enhance an effective metabolic response for appropriate postoperative tissue healing and organ function recovery.

This paper has limitations that we cannot neglect. First, the sample size of our meta-analysis—2900 patients- is not high, and further research and original studies are required to confirm these preliminary results. Despite improved outcomes in recent years, pancreatic resection is still associated with high mortality. Moreover, in recent years, body composition measurement using diagnostic CT scans has emerged to assess sarcopenia in oncology patients and the topic of this manuscript is not novel. Indeed, several studies and meta-analyses have evaluated the impact of sarcopenia on different clinical outcomes in PC patients. However, some of these studies included mixed patient cohorts regarding treatments (chemotherapy, palliative, surgery) and mixed CT-sarcopenia assessment methods. The diagnosis of low muscle mass in cancer patients varies widely not only in the choice of the slice but also on the studied area (psoas or skeletal muscle) and in the appropriate methods of comparison such as the sarcopenia cut-off values used, with or without sex-specific values. To date, a lack of consensus remains. The studies included in our meta-analysis used different cut-off values to dichotomize SMI. However, most studies used the cut-off values defined by Prado *et al*.^12^, 52.4 cm^2^/m^2^ for males and 38.5 cm^2^/m^2^ for females. Prado and colleagues determined the gender-specific L3 SMI cut-points which correlate with whole-body skeletal muscle mass and link to adverse outcomes in various populations including the intensive care unit, cancer, and liver disease patients. Considering that SMI has been also measured under differing conditions in terms of personal staff and equipment, the development of artificial intelligence may ameliorate accuracy and reliability in L3-CT-SMI assessment^50^. As regards RFS, although five studies including more than 1300 patients have assessed the RFS outcome, only two studies^21,26^ specified HR for RFS. Indeed, the other three studies^24,28,29^ provided only the curve of Kaplan–Meier. We tried to retrieve HR from the published data of Kaplan–Meier curves, using methods described by Parmar *et al*.^16^, but unfortunately, due to a lack of available statistical published data, it has not been possible to fulfil the objective of providing the forest plot for the estimation of the pooled HR for RFS. Moreover, a subgroup analysis accounting for the influence of confounders in the calculation of pooled HR for OS, such as tumour stage or absence of adjuvant chemotherapy would be appropriate but, the heterogeneity of studies was too high. Choi *et al*.^22^ selected as possible predictors such as age, sex, tumour size, nodal status, histologic differentiation, surgery type, resection status and performed a multivariable Cox analysis including all these predictors. However, the multivariate analysis of Okumara *et al*. identified high visceral fat/subcutaneous fat area ratio (VSR), low SMI, microvascular invasion, nodal metastasis, and absence of adjuvant chemotherapy as significant independent risk factors for mortality after PC resection^21^.

The major strength of this updated meta-analysis is its homogeneity in terms of the timing of assessment and reduction of muscle mass measurement. SMI measurement represents an easy nutritional assessment tool in the routine cancer course for clinicians since CT scan is analyzed for staging disease on the tumour board. We included the most recent published studies that exclusively measured L3-CT scan SMI in the specific timing preoperative period in order to identify a standardized method to assess nutritional status and predict clinical outcomes which can be used in clinical practice by the tumour board to identify vulnerable patients and propose an early personalized nutritional support.

## Conclusions

This meta-analysis demonstrated that preoperative L3-CT scan low SMI is associated with worse OS in pancreatic patients undergoing surgery. Preoperative low muscle mass measured by diagnostic CT scans affects more than half PC resectable patients. However, no significant associations between low muscle mass and postoperative outcomes are found. Since CT images are readily available for analysis and do not require additional cost, burden, or further radiation exposure to PC patients, CT-defined sarcopenia should be routinely assessed and discussed in a timely manner by a multidisciplinary tumour board, including clinical nutrition teams - physicians or dietitians - to care for more vulnerable patients, especially in the case of surgery.

## Ethical approval

Not applicable.

## Consent

Not applicable.

## Sources of funding

This study did not receive any specific grants from funding agencies in the public, commercial, or not-for-profit sectors.

## Author contribution

P.R.: study concept, writing the paper. M.C.: data collection. A.C.: data collection. G.P.: data analysis. M.P.: data interpretation. S.A.: supervision. G.T.: supervision. A.G.: review and editing. M.C.M.: review and editing. E.R.: study concept, review, and editing, coordination of the working group.

## Conflicts of interest disclosure

There are no conflicts of interest.

## Research registration unique identifying number (UIN)

This systematic review was registered in Prospero https://www.crd.york.ac.uk/prospero/ as number CRD42023438531. We confirm that the status of our registration with PROSPERO is “Review Completed not published”.

## Guarantor

Emanuele Rinninella.

## Data availability statement

Data are available within the article or its supplementary materials.

The authors confirm that the data supporting the findings of this study are available within the article [and/or] its supplementary materials.

## Provenance and peer review

Not commissioned, externally peer-reviewed.

## Supplementary Material

SUPPLEMENTARY MATERIAL
