# Supplementary material for: Preoperative low skeletal muscle mass index assessed using L3-CT as a prognostic marker of clinical outcomes in pancreatic cancer patients undergoing surgery: a systematic review and meta-analysis
Source: Int J Surg. 2023 Dec 11;110(10):6126–34. doi: 10.1097/JS9.0000000000000989 (PMC11486987; doi:10.1097/JS9.0000000000000989)
Supplement: SUPPLEMENTARY MATERIAL [file js9-110-6126-s003.docx]

Table S2. Search strategies

Pubmed 18.05.2023

| Search |  | Results |
| --- | --- | --- |
| #1 | pancreatectomy OR pancreaticoduodenectomy OR (pancreas AND surgery) OR  (Whipple’s AND procedure) OR (pancreatic AND leak) OR (postoperative AND pancreatic AND fistula) | 92,131 |
| #2 | sarcopenia OR (skeletal AND muscle AND index) OR SMI OR (muscle AND mass) | 15,200 |
| #3 | (pancreatic AND fistula) OR POPF OR (bile AND leak) OR complications OR morbidity OR (surgery AND outcomes) OR survival | 7,053,178 |
| #1 AND #2 AND #3 | | 57 |

Web of Science 18.05.2023

| Search |  | Results |
| --- | --- | --- |
| #1 | pancreatectomy OR pancreaticoduodenectomy OR (pancreas AND surgery) OR  (Whipple’s AND procedure) OR (pancreatic AND leak) OR (postoperative AND pancreatic AND fistula) | 59,886 |
| #2 | sarcopenia OR (skeletal AND muscle AND index) OR SMI OR (muscle AND mass) | 13,546 |
| #3 | (pancreatic AND fistula) OR POPF OR (bile AND leak) OR complications OR morbidity OR (surgery AND outcomes) OR survival | 8,296,413 |
| #1 AND #2 AND #3 Restricted to: clinical studies | | 48 |

Scopus 18.05.2023

| Search |  | Results |
| --- | --- | --- |
| #1 | pancreatectomy OR pancreaticoduodenectomy OR (pancreas AND surgery) OR  (Whipple’s AND procedure) OR (pancreatic AND leak) OR (postoperative AND pancreatic AND fistula) | 73,116 |
| #2 | sarcopenia OR (skeletal AND muscle AND index) OR SMI OR (muscle AND mass) | 18,963 |
| #3 | (pancreatic AND fistula) OR POPF OR (bile AND leak) OR complications OR morbidity OR (surgery AND outcomes) OR survival | 9,754,285 |
| #1 AND #2 AND #3 | | 71 |
