# Supplementary material for: Preoperative low skeletal muscle mass index assessed using L3-CT as a prognostic marker of clinical outcomes in pancreatic cancer patients undergoing surgery: a systematic review and meta-analysis
Source: Int J Surg. 2023 Dec 11;110(10):6126–34. doi: 10.1097/JS9.0000000000000989 (PMC11486987; doi:10.1097/JS9.0000000000000989)

Table S3: Excluded studies and reasons for exclusion

| **First Author** | **Year of publication** | **Reasons for exclusion** |
| --- | --- | --- |
| Wigmore | 1997 | BIA measurement |
| Barber | 2000 | BIA measurement |
| Wigmore | 2000 | BIA measurement |
| Bauer | 2005 | BIA measurement |
| House | 2008 | No SMI measurement |
| Tan | 2009 | Pretreatment SMI measurement |
| Aslani | 2010 | BIA measurement |
| Pelzer | 2010 | BIA measurement |
| Mathur | 2011 | No L3 CT scan |
| Dalal | 2012 | Pretreatment SMI measurement |
| Gaujoux | 2012 | No SMI measurement |
| Peng | 2012 | No SMI measurement |
| Vaisman | 2012 | Pretreatment DEXA measurement |
| Kirihara | 2013 | No SMI measurement |
| Falconer | 2014 | BIA measurement |
| Akahori | 2015 | No SMI measurement |
| Amini | 2015 | No SMI measurement |
| Choi | 2015 | Pretreatment SMI measurement |
| Cooper | 2015 | Pretreatment SMI measurement |
| Okumura | 2015 | PMI |
| Sur | 2015 | No SMI measurement |
| Wesseltoft-Rao | 2015 | Pretreatment SMI measurement |
| Pausch | 2016 | No SMI measurement |
| Park | 2016 | Pretreatment SMI measurement |
| Parsons | 2017 | Pretreatment SMI measurement |
| Carrara | 2017 | No SMI measurement |
| Delitto | 2017 | No SMI measurement |
| Ishii | 2017 | No SMI measurement |
| Benjamin | 2018 | Pretreatment SMI measurement |
| Bian | 2018 | Pretreatment SMI measurement |
| Jin | 2018 | Pretreatment L4 CT scan SMI measurement |
| Kurita | 2018 | Pretreatment SMI measurement |
| Sandini | 2018 | Pretreatment SMI measurement |
| Sugimoto | 2018 | No SMI measurement |
| Wagner | 2018 | No SMI measurement |
| Wu | 2018 | Pretreatment SMI measurement |
| Basile | 2019 | Pretreatment SMI measurement |
| Kurita | 2019 | Pretreatment SMI measurement |

Abbreviations: BIA: Body Impedance Analysis, CT, computing Tomography, DEXA: Dual-energy X-ray absorptiometry, PMI: Psoas muscle index, HU: Hounsfield Units, SMI: Skeletal Muscle Index.

Supplementary Figure 1 Funnel plots for examination of publication bias with 95% confidence intervals and *p-values* for Egger linear regression tests for the outcomes: (A) Overall Complications (B) Major Complications (C) Pancreatic Fistula (D) Operating Time (E) Peri-Operative Mortality (F) Overall Survival

**
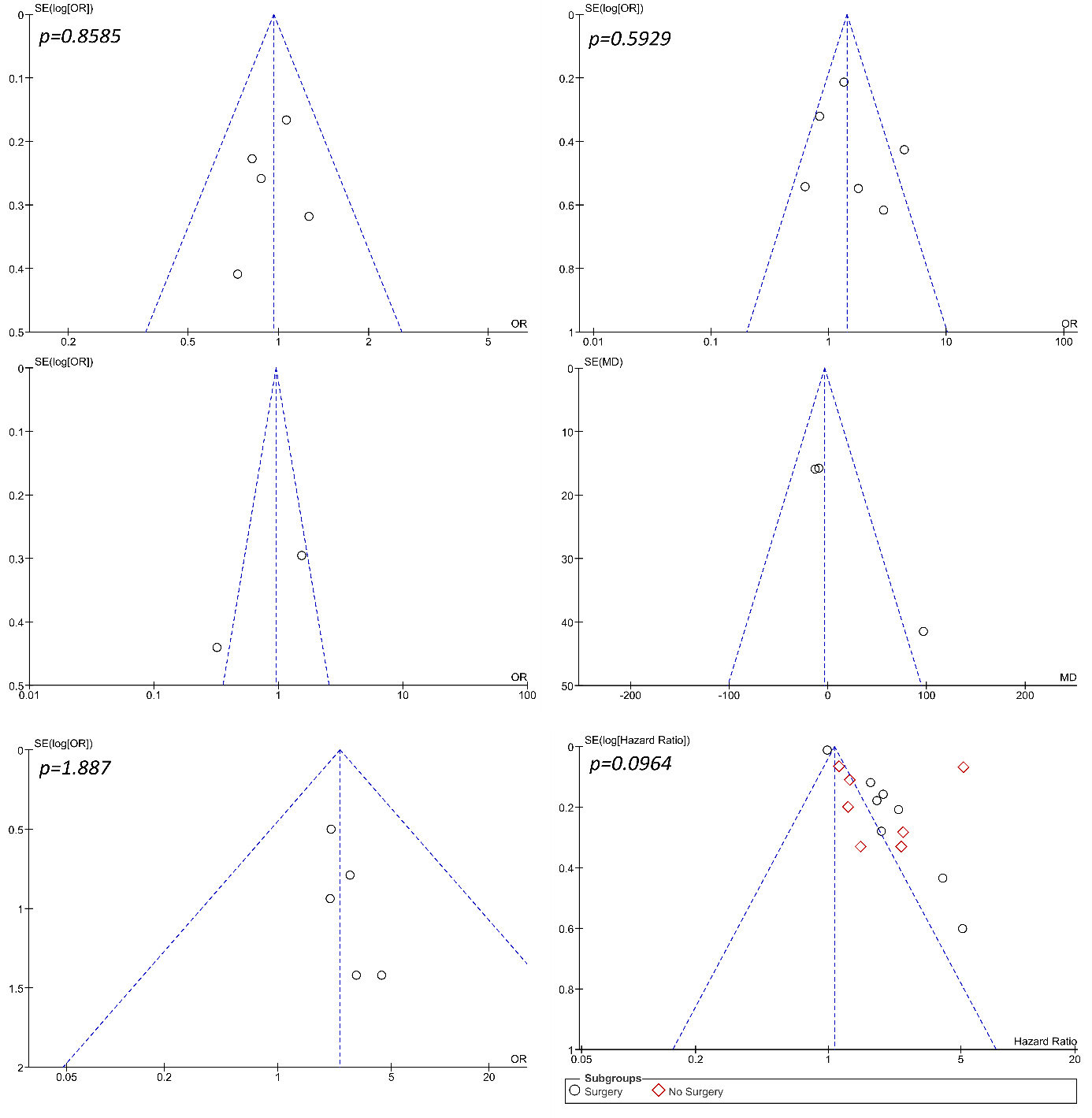
**

Supplementary Figure 2 Summary meta-analysis of studies reporting impact of pre-operative sarcopenia on operating time in patients undergoing gastrectomy
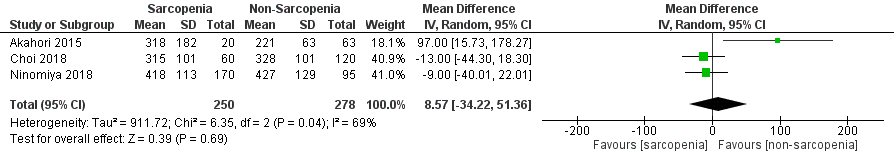


Supplementary Figure 3 Summary meta-analysis of studies reporting impact of pre-operative sarcopenia on blood loss in patients undergoing pancreatic resection
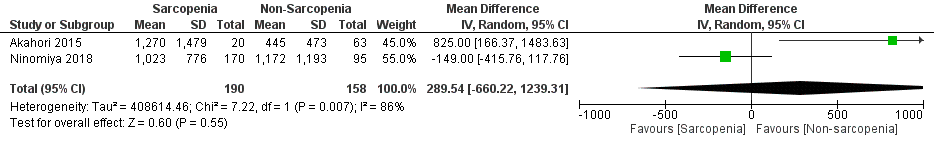

Supplement: SUPPLEMENTARY MATERIAL [file js9-110-6126-s004.docx]
