# Supplementary material for: Preoperative low skeletal muscle mass index assessed using L3-CT as a prognostic marker of clinical outcomes in pancreatic cancer patients undergoing surgery: a systematic review and meta-analysis
Source: Int J Surg. 2023 Dec 11;110(10):6126–34. doi: 10.1097/JS9.0000000000000989 (PMC11486987; doi:10.1097/JS9.0000000000000989)

Figure S1. Funnel plots (a) overall survival unadjusted (b) overall survival adjusted (c) POPF (d) morbidity (e) length of hospital stay

(a)


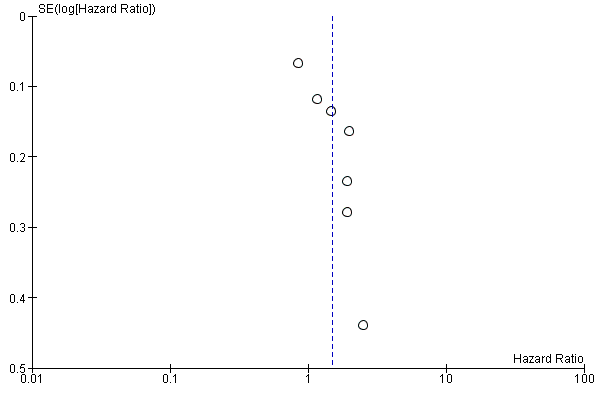


(b)


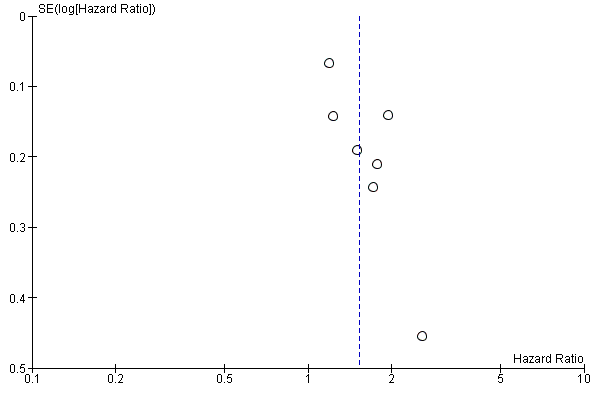


(c)


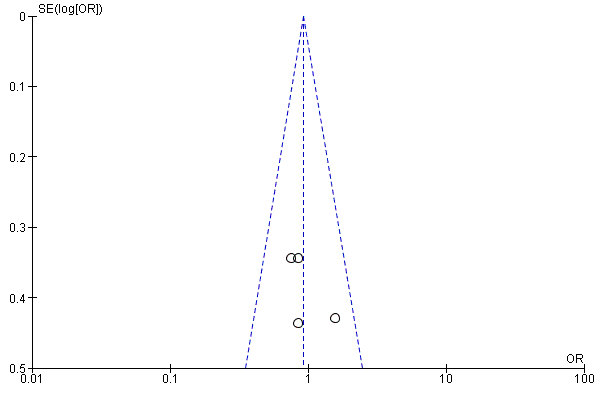


(d)


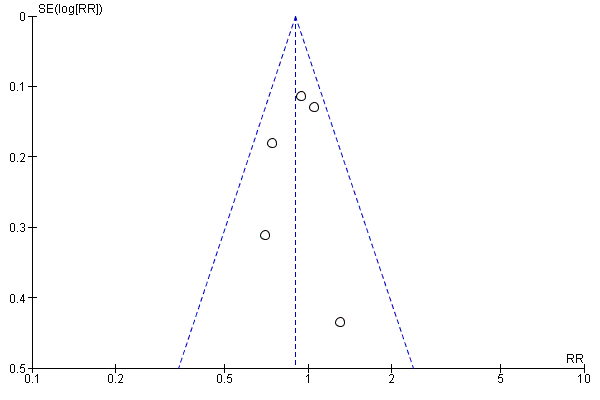


(e)


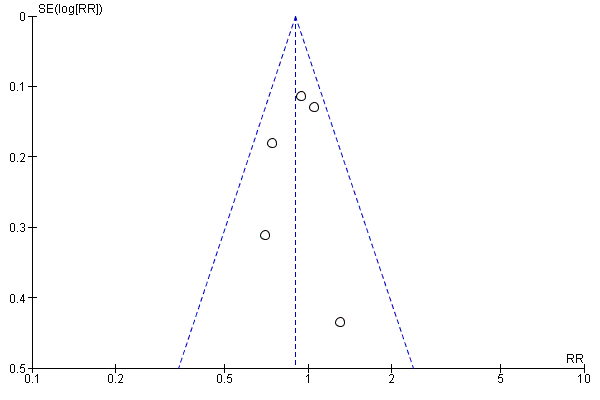

Supplement: SUPPLEMENTARY MATERIAL [file js9-110-6126-s005.docx]
