# Supplementary material for: Preoperative low skeletal muscle mass index assessed using L3-CT as a prognostic marker of clinical outcomes in pancreatic cancer patients undergoing surgery: a systematic review and meta-analysis
Source: Int J Surg. 2023 Dec 11;110(10):6126–34. doi: 10.1097/JS9.0000000000000989 (PMC11486987; doi:10.1097/JS9.0000000000000989)
Supplement: SUPPLEMENTARY MATERIAL [file js9-110-6126-s006.docx]

Table S4: Quality assessment of retrospective studies

| **Author,**  **Year** | **Is the case definition adequate** | **Represen-tativeness of the cases** | **Selection of Controls** | **Definition of Controls** | **Comparability of cases and controls on the basis of the design or analysis** | **Ascertainment of exposure** | **Same method of ascertainment for cases and controls** | **Non-Response rate** | **NOS**  **score** |
| --- | --- | --- | --- | --- | --- | --- | --- | --- | --- |
| Pecorelli et al, 2016 | ***** | ***** | ***** | ***** | ****** | ***** | ***** | ***** | 8 |
| Ninomiya et al, 2017 | ***** | ***** | ***** | ***** | ****** | ***** | ***** | ***** | 8 |
| Okumura et al, 2017 | ***** | ***** | ***** | ***** | ****** | ***** | ***** | ***** | 8 |
| Choi et al, 2018 | ***** | ***** | ***** | ***** | ****** | ***** | ***** | ***** | 8 |
| Sugimoto et al, 2018 | ***** | ***** | ***** | ***** | ***** | ***** | ***** | ***** | 8 |
| Gruber et al. 2019 | ***** | ***** | ***** | ***** | ***** | ***** | ***** | ***** | 8 |
| Ryu et al, 2020 | ***** |  | ***** | ***** | ***** | ***** | ***** | ***** | 7 |
| Rom et al, 2021 | ***** | ***** | ***** | ***** | ***** | ***** | ***** | ***** | 8 |
| Menozzi, 2023 | ***** | ***** | ***** | ***** | ****** | ***** | ***** | ***** | 8 |
| Shen, 2023 | ***** | ***** | ***** | ***** | ***** | ***** | ***** | ***** | 8 |
| Masuda, 2023 | ***** | ***** | ***** | ***** | ***** | ***** | ***** | ***** | 8 |
